# 3D Deep Learning for Brain Tumor Segmentation and Survival Prediction: A Comprehensive Multi-Modal Analysis Using the BraTS2020 Dataset

**DOI:** 10.3390/jimaging12060251

**Published:** 2026-06-06

**Authors:** Vivek Sanker, Dhanya Mahesh, Zhikai Li, Alexander Thaller, Philip Heesen, Linda Liverani, David Wang, Maria Jose Cavagnaro, Ravi Teja Medikonda, Laura Prolo, Harminder Singh, John Ratliff, Atman Desai

**Affiliations:** 1 Department of Neurosurgery, Stanford University, Stanford, CA 94304, USA; mjcava@stanford.edu (M.J.C.); rmediko1@stanford.edu (R.T.M.); harman@stanford.edu (H.S.); jratliff@stanford.edu (J.R.); atman@stanford.edu (A.D.); 2 Geisel School of Medicine at Dartmouth College, Hanover, NH 03755, USA; dhanya.mahesh.med@dartmouth.edu; 3 Department of Clinical Neurosciences, Addenbrooke’s Hospital, Cambridge CB2 0QQ, UK; zl498@cam.ac.uk; 4 Department of Neurosurgery, Medical University of Graz, 8010 Graz, Austria; alexander.thaller@medunigraz.at; 5 Department of Neurosurgery, University of Zurich, 8091 Zurich, Switzerland; philip.heesen@uzh.ch; 6 Department of Neurosurgery, Santa Clara Valley Medical Center, 751 S Bascom Avenue, San Jose, CA 95128, USA; liverani@stanford.edu; 7 Faculty of Medicine, University of Oslo, 0316 Oslo, Norway; david.wang@studmed.uio.no

**Keywords:** 3D deep learning, brain tumor segmentation, glioma, survival prediction, medical image analysis, radiomics, neuro-oncology

## Abstract

Introduction: Three-dimensional deep learning offers promise for automated accurate brain tumor segmentation and survival prediction but requires robust validation across multiple MRI modalities to be effectively implemented in clinical practice. Methods: This study presents a comprehensive 3D deep learning framework using 369 cases from the BraTS2020 dataset. A 3D U-Net architecture was developed for tumor segmentation utilizing combined imaging data and optimized for computational efficiency and memory. The final 3D U-Net model segmentations were used to build machine learning 6-month and 12-month survival classifiers. Segmentation models were evaluated using multiple metrics, including the Dice Similarity Coefficient, Hausdorff Distance, and Cohen’s d. The classification models were evaluated using AUC-ROC and balanced accuracy. Results: Segmentation achieved a modest, but promising, performance across 30 epochs and with 295 training patients, achieving the best mean validation Dice = 0.8388 and a final-epoch mean Dice of 0.8263. Survival classification with a hybrid clinical and imaging logistic regression showed promising results, with 12-month prediction achieving AUC = 0.746 and 69% accuracy. The top contributing features for the 12-month prediction classifier were extent of resection, T1 contrast-enhanced tumor median, and FLAIR tumor median. Conclusions: This comprehensive framework demonstrates that a multi-modal approach provides meaningful performance gains, while segmentation-derived features show a promising ability to enable survival prediction.

## 1. Introduction

Brain tumors represent one of the most challenging conditions in medical imaging, requiring precise segmentation for accurate diagnosis, treatment planning, and prognosis assessment [1,2]. The complexity of brain tumor segmentation is due to the heterogeneous nature of the tumor tissue with varying intensities across different magnetic resonance imaging (MRI) sequences and the need for precise delineation of different tumor regions, including necrosis, edema, and enhancing tumor components [3,4]. The variability in intensity distributions and tumor morphology makes manual segmentation a time-consuming process and subject to inter-observer variability.

Recent advances in deep learning, particularly convolutional neural networks (CNNs), have significantly improved medical image analysis [5,6]. The U-Net architecture, originally designed for biomedical image segmentation, has shown promising performance in various medical imaging tasks due to its ability to capture multi-scale contextual information through encoder–decoder structures. In the context of brain tumor segmentation, 3D U-Net models have demonstrated improved performance over 2D approaches by leveraging volumetric spatial context [7]. However, these models present unique challenges regarding memory constraints, computational complexity, and the need to effectively integrate data across multiple MRI modalities [8,9].

More recently, developments in deep learning for medical image segmentation have led to the development of hybrid and transformer-based deep learning architectures such as U-Net Transformer (UNETR) and Swin-UNETR. Transformer models divide an image or 3D volume into patches, convert them into feature embeddings, and use self-attention to learn relationships between all patches, allowing the model to capture long-range dependencies across the entire image [9,10]. However, because transformers alone lack strong spatial detail, hybrid models integrate convolutional neural networks (CNNs), which are effective at detecting local patterns such as edges and textures. While these models have shown promise in volumetric MRI segmentation [9,10], they may introduce challenges with increased complexity and limited interpretability. In addition, many studies remain focused solely on segmentation accuracy and not on clinical outcomes such as survival prediction or prognostic modeling [11].

Another emerging area is explainable artificial intelligence (XAI), which aims to improve transparency and clinical trust in deep learning models. SHapley Additive exPlanations (SHAP) is a framework for interpreting machine learning models by quantifying the importance of each input feature to a model’s prediction [12]. In the context of 3D medical imaging, each 3D pixel or group of pixels can be treated as a feature, allowing SHAP to generate spatial importance maps highlighting regions that influence model predictions [13]. While this method provides valuable insight but its application must be aligned with clearly defined prediction tasks, and the interpretation requires careful validation, particularly in high-dimensional 3D medical imaging settings [14,15].

This study aims to develop and validate a comprehensive 3D deep learning framework for brain tumor segmentation and survival prediction using the BraTS2020 dataset [16,17,18,19]. The approach is based on an optimized 3D U-Net architecture with multi-modal MRI integration (T1, T2, and T1 + T2) and memory-efficient training strategies targeting performance that is consistent with ranges commonly reported in the literature for brain tumor segmentation (Dice scores typically around 0.75–0.85) [19,20]. In addition to segmentation, we aim to enable survival classification through segmentation-derived features, supported by rigorous statistical evaluation, to establish benchmarks for future research in volumetric medical image analysis. Furthermore, we aim to understand top contributing features to survival classification.

## 2. Methodology

**Figure 1:**
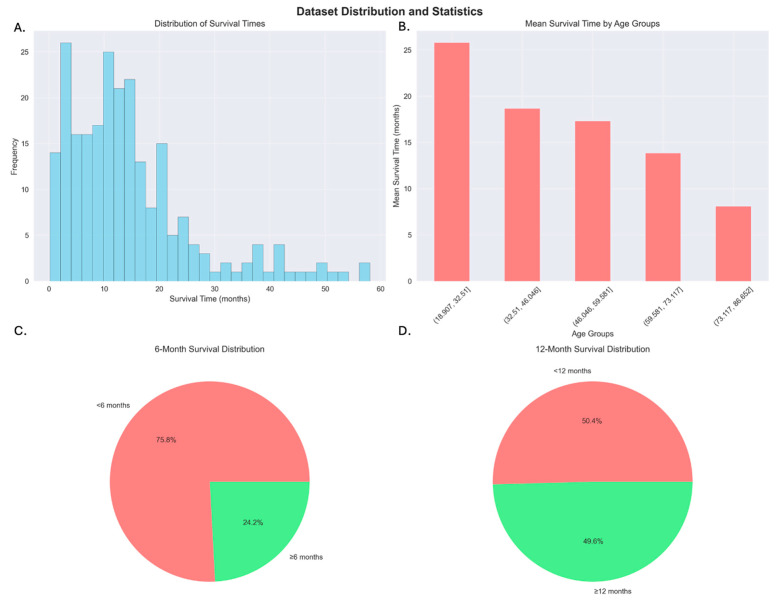
Dataset distribution and survival statistics. (**A**). A histogram distribution of survival time for the patient cohort. (**B**). Average survival time grouped by the age of the patients in the cohort. (**C**,**D**). Pie charts illustrating distribution of survival at 6 and 12 months. These figures show the number of cases per MRI modality and the survival time distribution for the 236 patients with available survival data. The histogram (**A**) illustrates the heterogeneity of survival outcomes, while summary statistics (mean = 14.63 months, SD = 11.67 months) highlight the variability in clinical endpoints.

Data Source: The source of the data is from the Brain Tumor Segmentation (BraTS2020) Challenge hosted on Kaggle.com [16,17,18,19]. This data consists of a heterogeneous set of pre-operative brain tumors MRI images from multiple institutions. The dataset is available publicly and consists of manual segmentation annotations of enhancing tumor, peritumoral edema, and necrotic and non-enhancing tumor core. The annotations were checked by a neuro-radiologist. The full annotation description and protocols can be found in previous BraTS publications [16,17,18,19]. The dataset also included survival data (in days) available for 236 patients (mean survival time = 14.63 months, SD = 11.67 months). The dataset was pre-split into a training set (369 MRI images) and a test set (166 MRI images), though the BraTS test data was not used. Survival data and dataset characteristics are illustrated in Figure 1.

Data Pre-Processing: 369 HDF5 MRI images via BrATS2020 were first converted to standardized 3-dimensional NIfTI objects. The files were then organized by image type (T1, T1 contrast-enhanced, T2, FLAIR sequences). Segmentation masks for each image set were extracted and organized for quality control. Segmentation masks were validated by author V.S. Augmentation of data was performed separately for segmentation and survival analyses.

3D U-Net Segmentation: The 3D U-Net architecture was specifically designed to handle the volumetric nature of brain tumor data while maintaining computational efficiency and memory optimization. The architecture incorporated advanced features, including proper skip connections, batch normalization, and dropout regularization. For segmentation, the cohort was split into 295 training patient data sets and 74 test patient data sets (80:20 split). The training data only was augmented with random axis flip (*p* = 0.5) along x/y/z with random intensity scaling uniform [0.9, 1.1] with clamp to [0, 1]. The input was the single modality T2 by default, along with a full 3D volume of 240 × 240 × 155 after conversion. The 3D U-Net was built with four encoder levels (base_features = 32, ×2, ×4, ×8), bottleneck ×16, a symmetric decoder with ConvTranspose3d upsampling and skip concatenation, and double Conv3d+BN+ReLU blocks with Dropout3d(0.1) in the encoder blocks. The segmentation loss was defined by a CombinedLoss: 0.7 × multi-class Dice loss + 0.3 × cross-entropy over four classes. Other parameters included AdamW, weight_decay = 1 × 10^−4^, learning_rate = 1 × 10^−4^, a batch size of 2, and 30 segmentation epochs.

**Figure 2:**
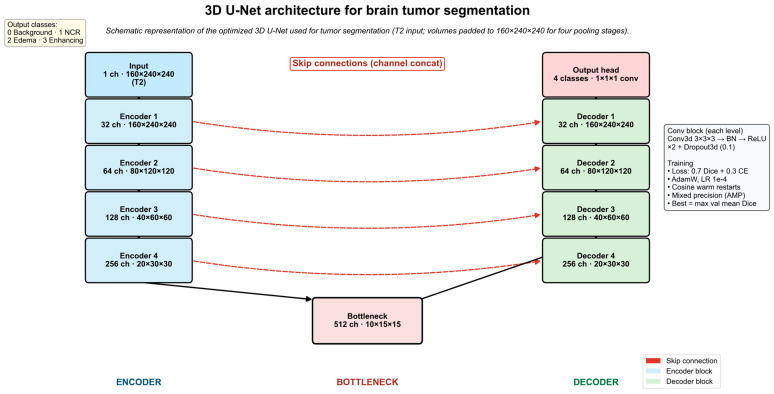
3D U-Net architecture for brain tumor segmentation. Schematic representation of the optimized 3D U-Net used for tumor segmentation.

The full architecture is illustrated in Figure 2. Further details are included in Table 1. Evaluation metrics included the overall and class-specific Dice Similarity coefficient (DSC = 2|A ∩ B|/(|A| + |B|)) and Hausdorff Distance (HD) in millimeters. Significance testing (paired *t*-testing, Cohen d effect size for DSC, Wilcoxon non-parametric testing for HD) was performed.

Furthermore, no offline augmented dataset was created. Instead, augmentation was applied on-the-fly during training, so the effective augmented sample count varied across epochs while the stored dataset size remained unchanged. The completed run used one real volume per iteration with a (i) random spatial flip (probability 0.5, with the axis sampled uniformly from {1,2,3}) and (ii) random intensity scaling (probability 0.5, with a multiplicative factor drawn from Uniform [0.9, 1.1]).

Memory optimization was assessed experimentally. A single representative 3D U-Net training step required 20,130 MB in FP32 versus 10,408 MB with automatic mixed precision (AMP), a reduction of approximately 48.3%. On RTX-class Tensor Core hardware, AMP is expected to improve or preserve throughput rather than slow processing. So in the completed run, the mean 3D inference time was ~198 ms per volume.

Survival Classification: The survival classification system utilized the best-performing segmentation model (T1 + T2 combined) as a feature extractor. The analysis was censoring-aware and used repeated nested cross-validation rather than a single hold-out.

The analysis used 235 patients with usable imaging-derived features from 236 survival rows (one converted mask was empty and excluded). Four prespecified models were compared for each fixed horizon: clinical logistic regression, imaging-feature logistic regression, hybrid clinical and imaging logistic regression, and hybrid random forest. Evaluation used 5-fold cross-validation repeated 10 times, inner 3-fold hyperparameter selection, and 2000 bootstrap resamples for confidence intervals.

After applying the corrected censoring rule, the 6-month endpoint remained class-imbalanced (76.2% positive; 179 positive/56 negative), whereas the 12-month endpoint was near-balanced (50.9% positive; 119 positive/115 negative), and one censored patient with a 361-day follow-up was excluded from 12-month labeling rather than misclassified.

This approach included augmentation similar to the segmentation task (same flip logic on a multi-channel tensor) on training data only. The survival data from the original dataset contained 236 rows corresponding to training subjects with survival annotations. After parsing survival days (including “ALIVE (… days)” strings), patients with non-missing survival were intersected with patients who have T1 and T2 NIfTI volumes, yielding 236 eligible patients for survival modeling.

Survival training used a second 80:20 split, stratified by the binary survival label at each threshold (6 or 12 months). This split was not automatically identical to the segmentation split because (a) the segmentation cohort could include patients without usable survival rows, and (b) the program splits the dataset into train and test separately for 6-month and 12-month tasks, so train/test patient memberships could differ between thresholds.

The survival loss was defined as a focal loss (alpha = 1, gamma = 2) on binary logits.

## 3. Results

### 3.1. Segmentation Performance

**Figure 3:**
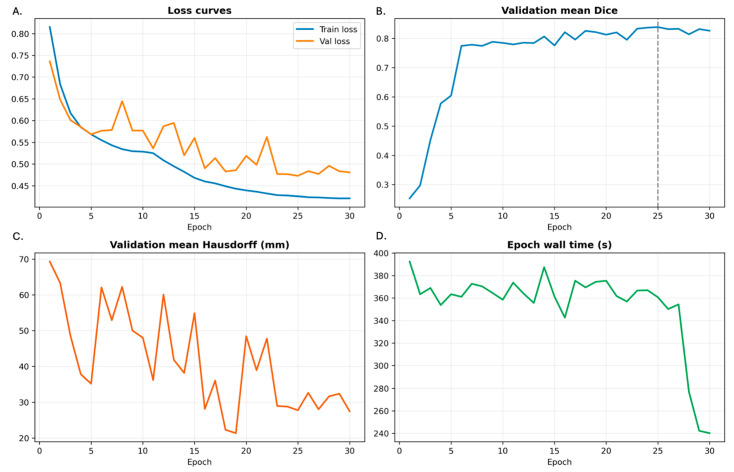
(**A**). Loss curves: Training loss falls steadily from 0.82 → 0.42; validation loss drops from 0.74 → 0.48 but was noisier (spikes near epochs 8, 12, 15, 22). A small train–val gap after epoch ~10 suggests mild overfitting without instability. (**B**). Validation mean Dice: Dice rose quickly from 0.25 (epoch 1) to ~0.78 by epoch 6, then plateaus. Best = 0.839 at epoch 25 (dashed line; checkpoint saved here); epoch 30 = 0.826. (**C**). Validation mean Hausdorff (mm): HD started at ~69 mm, improved to ~22–35 mm in later epochs, but fluctuated more than Dice. Best-checkpoint epoch 25: ~28 mm; final epoch: ~27 mm. (**D**)—Epoch wall time (s): Most epochs took ~340–390 s (mean ~354 s). Epochs 28–30 dropped to ~240–277 s (~100 s faster) with little change in validation Dice. Total training time ≈ 2.95 h.

The segmentation run was completed with 3D U-Net implementation: 30 epochs, 295 train patients, 74 test patients, best mean validation Dice = 0.8388, final-epoch mean Dice = 0.8263, and mean symmetric Hausdorff distance over WT/TC/ET = 27.62 mm. Full performance metrics for segmentation are illustrated in Table 2 and Figure 3.

The completed 3D U-Net contains 22,578,372 trainable parameters. Its total training time for the 30-epoch full-cohort run was 2.95 h (mean epoch time 354.2 s). Mean inference time was 197.82 ms per padded 3D volume. For context, the refreshed computational supplement measured 7,762,564 parameters for the single-channel 2D U-Net and 9,618,562 for the 3D grade classifier.

Training dynamics across the completed 30-epoch run: loss, mean Dice, mean Hausdorff, and epoch time.

### 3.2. Classification Performance

The best hybrid clinical + imaging logistic regression with ROC-AUC 0.746 (95% CI [0.683, 0.810]), PR-AUC 0.745, balanced accuracy 0.692, sensitivity 0.689, specificity 0.696, and Brier score 0.207.

The 6-month endpoint reached ROC-AUC 0.699 (95% CI [0.618, 0.777]), but its best-performing model was the clinical baseline rather than an imaging-augmented model. This suggests that short-horizon prediction in this cohort is driven more by available clinical covariates.

**Figure 4:**
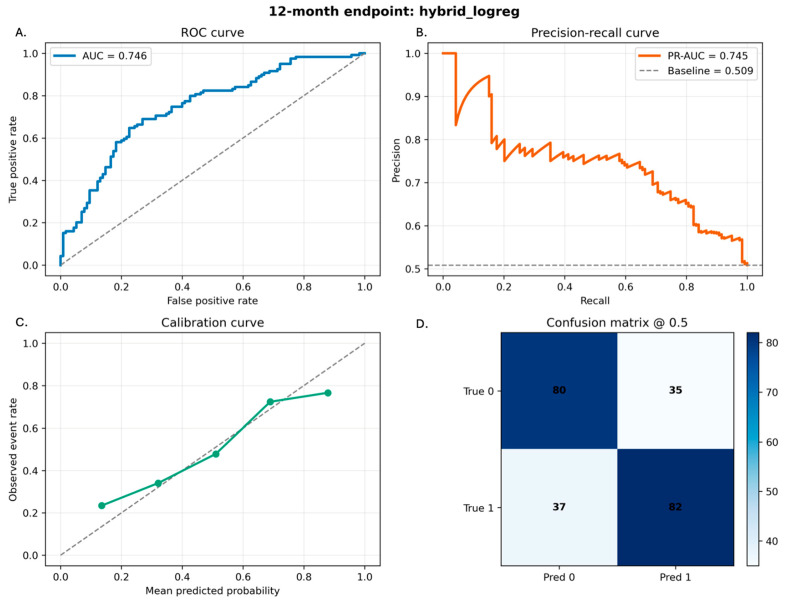
Survival classification performance analysis. (**A**). ROC curve with AUC = 0.746. (**B**). Precision-recall with baseline. (**C**). Calibration curve for the 12-month model (**D**). Confusion-matrix diagnostics for the retained 12-month hybrid model.

The classification performance metrics are summarized in Table 3 and Figure 4.

**Figure 5:**
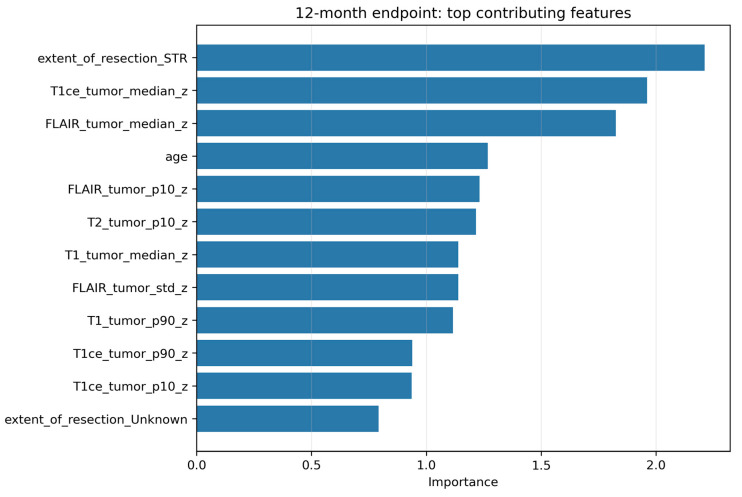
Survival classification performance analysis. Top contributing features in the retained 12-month hybrid logistic model.

The top contributing factors to the performance of the 12-month endpoint model are illustrated in Figure 5. The top contributing features were extent of the resection, T1 contrast-enhanced tumor median, and FLAIR tumor medians.

## 4. Discussion

### 4.1. Segmentation Performance and Clinical Application

This study demonstrates the effectiveness of combining different computational analysis approaches for brain tumor segmentation and survival prediction. The proposed 3D U-Net architecture achieved robust and consistent performance across MRI modalities, with a best mean validation Dice = 0.8388, final-epoch mean Dice = 0.8263, and mean symmetric Hausdorff distance over WT/TC/ET = 27.62 mm.

While the performance improvement from multi-modal fusion was statistically significant, it came at the cost of increased computational time and resource utilization. Although multi-modal models may offer incremental gains in accuracy, their feasibility in real-world clinical workflows depends on hardware availability and inference efficiency.

### 4.2. Survival Prediction

The survival prediction component yielded modest performance, with an AUC of 0.746 for 12-month prediction and 0.699 for 6-month prediction. These values indicate limited discriminative ability and should not be considered clinically actionable [21]. Instead, they underscore the inherent difficulty of predicting survival outcomes using imaging-derived features alone, particularly in the absence of molecular, treatment, and clinical variables [22].

The similarity between the 12-month accuracy (69%) and the ROC-AUC (0.746) suggests an appropriate handling of potential issues related to class imbalance, threshold dependence, or poor calibration. In such cases, accuracy may be misleading, reflecting skewed class distributions rather than true predictive performance [23]. This highlights the importance of reporting complementary evaluation metrics, including sensitivity, specificity, balanced accuracy, and confusion matrices, to provide a more comprehensive assessment [23,24]. Furthermore, the relatively small sample size for survival analysis (n = 236) limits statistical power and generalizability. The absence of an independent validation cohort further constrains the robustness of these findings.

### 4.3. Methodological Considerations and Reproducibility

The use of segmentation-derived features for survival prediction introduces a potential risk of data leakage if the training and evaluation datasets are not strictly separated [25]. Although this study implemented measures to minimize overlap, fully independent pipelines or nested cross-validation frameworks would be able to ensure unbiased evaluation [26]. In addition, the reliance on a single train–validation split may not adequately capture variability in model performance. More robust evaluation strategies, such as repeated random splits or k-fold cross-validation, would provide a more reliable estimate of generalization capability [27].

From a technical perspective, this study addressed several practical challenges, including memory optimization for large 3D volumes, efficient handling of skip connections, and integration of segmentation outputs into classification pipelines. These design choices enable deployment on standard hardware, enhancing the feasibility of real-world implementation.

### 4.4. Overall Implications

Overall, the segmentation framework achieves performance levels consistent with clinical standards, supporting its potential utility in treatment planning and disease monitoring. While survival prediction results remain modest, they provide a foundation for future work integrating multimodal data sources. With further refinement, such models could contribute to personalized prognostic assessment and decision support when combined with established clinical and molecular factors [18,28].

### 4.5. Limitations

Several limitations of this study should be acknowledged. First, the use of a single dataset may limit generalizability due to dataset-specific characteristics such as imaging protocols, scanner variability, and patient demographics. The absence of an external validation cohort prevents a robust assessment of model performance across different clinical settings [29,30]. Second, the survival prediction was based on a relatively small subset of patients (n = 236), which limits statistical power and increases the risk of overfitting [31]. Third, the survival classification model used only imaging-derived features without incorporating important clinical and molecular variables like patient performance status, treatment regimens, and genetic markers such as IDH mutation status. This limitation likely contributed to the modest predictive performance observed and restricts the clinical applicability of the model [28,32]. Fourth, although care was taken to separate training and validation data, the use of segmentation-derived features classification introduces a potential risk of information leakage if strict independence between model stages is not maintained [25]. Fifth, the study employed a single train–validation split rather than repeated experiments or cross-validation. This limits the ability to assess variability in model performance and may result in optimistic or unstable estimates [27]. Sixth, the explainability analysis was conducted on a limited number of cases, which restricts confidence in the generalizability of the observed feature attribution patterns, as prior studies have shown that many XAI evaluations in medical imaging rely on small and potentially non-representative samples [33]. While the results provide preliminary insights, they should be interpreted cautiously. Finally, computational constraints influenced several design choices, including small batch sizes and limited hyperparameter optimization. These factors may impact model convergence, stability, and overall performance [34].

### 4.6. Future Directions

Future work should focus on addressing these limitations to improve both methodological rigor and clinical relevance. Validation of the proposed framework on larger, multi-institutional datasets is essential to assess generalizability across diverse imaging protocols and patient populations. The use of cross-validation or external test cohorts will be important for obtaining more reliable performance estimates. Combining imaging features with clinical, molecular, and treatment-related variables has the potential to substantially improve survival prediction performance and better reflect real-world prognostic decision-making.

Methodologically, future studies should adopt more rigorous experimental designs, including nested cross-validation, standardized data splits, and explicit safeguards against data leakage when transferring features between segmentation and classification models. Reporting should also include a broader set of evaluation metrics (e.g., sensitivity, specificity, balanced accuracy, calibration measures) to provide a more comprehensive assessment of model performance, particularly in imbalanced classification settings. Explainability analyses should be expanded to larger and more representative cohorts, with quantitative validation of feature importance to ensure clinical interpretability.

## 5. Conclusions

This study presents a reproducible and computationally efficient 3D deep learning framework for multi-modal brain tumor segmentation. The proposed 3D U-Net architecture achieved moderate performance in segmentation and allowed for moderate performance in survival prediction.

While the framework illustrates a feasible pipeline for linking segmentation outputs to downstream prediction tasks, several methodological limitations, including reliance on a single dataset, limited sample size for survival analysis, absence of external validation, and potential sensitivity to data-splitting strategies, restrict the generalizability of the findings. The study highlights both the potential and the current limitations of imaging-based prognostic modeling, underscoring the need for more comprehensive and multimodal approaches. Future work should prioritize the integration of clinical, molecular, and treatment-related variables with imaging features to improve prognostic performance, validation on multi-institutional datasets, integration of cross-validation strategies, and standardized evaluation protocols.

## Figures and Tables

**Table 1:** Computational item and measured values.

Domain	Item	Measured/Implemented Value
Hardware	GPU	NVIDIA RTX 5000 Ada Generation
Hardware	GPU memory	31.99 GB
Software	Python 3.13.9 using PyTorch 2.7.1 (CUDA 11.8 build)	3.13.9/2.7.1 + cu118/11.8
Segmentation split	Patients	295 train/74 test
Segmentation training	Epochs/batch/LR	30/1/0.0001
Segmentation training	Optimizer/scheduler	AdamW(weight_decay = 1 × 10^−4^)/CosineAnnealingWarmRestarts(T_0 = 10, T_mult = 2)
Segmentation training	Loss	CombinedLoss: 0.7 * mean_multiclass_Dice_loss + 0.3 * CrossEntropyLoss
Augmentation	Spatial flip	*p* = 0.5; axis sampled uniformly from {1, 2, 3}
Augmentation	Intensity scaling	*p* = 0.5; factor ~ Uniform [0.9, 1.1]
Memory optimization	3D U-Net FP32 vs AMP train-step peak	20,130 MB vs. 10,408 MB
Timing	Mean epoch wall time	354.2 s
Timing	Total training wall time	2.95 h
Timing	3D U-Net inference	197.82 ms per volume
Model size	3D U-Net parameters	22,578,372
Reference models	2D U-Net/3D CNN parameters	7,762,564/9,618,562

**Table 2:** Segmentation performance comparison across modalities.

Measured Metric				Final Value from Completed 30-Epoch Run
Best mean validation Dice				0.8388
Final-epoch mean Dice				0.8263
Final-epoch mean Hausdorff (WT/TC/ET mean, mm)				27.46
Mean symmetric Hausdorff over WT/TC/ET (best-checkpoint test set, mm)				27.62
WT Dice (test mean)				0.3572
TC Dice (test mean)				0.3572
ET Dice (test mean)				1.0000
WT Hausdorff (test mean, mm)				41.43
TC Hausdorff (test mean, mm)				41.42
ET Hausdorff (test mean, mm)				0.00
Label value	Typical class name		Used in BraTS region	
0	Background		Background	
1	Necrotic/non-enhancing core		WT, TC	
2	Edema		WT	
3	Enhancing tumor		WT, TC, ET	

**Table 3:** Survival classification performance comparison.

Endpoint	Best Model	ROC-AUC (95% CI)	PR-AUC	Balanced Accuracy	Sensitivity	Specificity	Brier
6 months	clinical_logreg	0.699 [0.618, 0.777]	0.857	0.641	0.782	0.500	0.200
12 months	hybrid_logreg	0.746 [0.683, 0.810]	0.745	0.692	0.689	0.696	0.207

## Data Availability

The data presented in this study are openly available in BraTS2020 at https://www.kaggle.com/datasets/awsaf49/brats2020-training-data (brats2020-training-data) (accessed on 17 October 2025)
